# Facemask Alternatives in Veterinary Medicine in the Context of COVID-19 Shortages

**DOI:** 10.3389/fvets.2020.00561

**Published:** 2020-08-27

**Authors:** Abigail Gavra Michaelson Purens

**Affiliations:** ^1^University of Minnesota College of Veterinary Medicine, St. Paul, MN, United States; ^2^Biological Resources Laboratory, University of Illinois at Chicago, Chicago, IL, United States

**Keywords:** cloth mask, facemask shortage, filtering face piece respirators, pandemic (COVID-19), personal protective equipment, SARS-CoV-2, surgical mask, veterinary medicine

## Abstract

The COVID-19 pandemic has caused a widespread shortage of facemasks and other personal protective equipment in veterinary medicine without clear, research-based guidance on alternatives to FDA-certified disposable surgical masks. In the absence of detailed veterinary research, an in-depth review of the human medical literature was conducted to evaluate the viability of reusable, sterilizable cloth, medical textile, or other material alternatives that may be quickly manufactured and used by veterinarians. The results at the time of publication support the AVMA, CDC, and WHO recommendations to extend use, reuse, and resterilize facemasks before considering using a homemade facemask. Pending further research, or until and unless the FDA certifies a reusable homemade mask or design, the substitution of homemade masks for FDA-certified surgical masks should only be considered as a last resort. Most homemade masks are not suitable replacements for N95 FFRs. If a homemade facemask must be made, the following materials and testing guidelines are suggested:

- densely woven cotton fabric (≥270 thread count), medical textile, or other impermeable, breathable material that can be laundered and resterilized
- make pleated or fitted pocket style facemasks to maximize fit
- make masks with two sets of ties, not elastics, to endure laundering and autoclaving
- internal wire or fabric tape may be used to mold masks over the nose
- filter material should be designed for use near mucus membranes, such as facial tissue or paper towel
- if possible, fit test homemade masks against an FDA-certified surgical mask as a control
- if higher filtration efficiency is required, test according to the FDA Enforcement Policy for Face Masks and Respirators During the Coronavirus Disease (COVID-19) Public Health Emergency
- Maintain enough masks to change as frequently as one would change disposable surgical masks to maintain appropriate hygiene.

## Introduction

With the introduction and pandemic spread of SARS-CoV-2 through the global human population, supply chains for commonly used, FDA-certified disposable surgical masks and N95 filtering facepiece respirators (FFRs) to protect medical personnel and lay people from the spread of this respiratory virus have been severely depleted (1). As all supplies of these masks and other personal protective equipment (PPE) are being redirected toward human medical professionals on the front lines of this pandemic, our use of these masks in veterinary medicine is called into question. Moreover, if viable reusable options exist, it may in fact behoove certain segments of veterinary medicine to continue using those options in their practice going forward after the current pandemic crisis has resolved. In the absence of any single resource currently addressing these concerns, particularly as they affect veterinary medicine, this review surveys technology and research on different types of respirator and surgical masks and potential replacements, with an eye to making recommendations on their use in veterinary practice. In the absence of relevant veterinary literature, this review follows the precedent of focusing on the human medical literature, to be adapted for veterinary use.

## Background

### Current Use of Facemasks as PPE in Veterinary Medicine

Regarding the transmission of respiratory pathogens, including SARS-CoV-2, the World Health Organization (WHO) defines droplets as pathogen-containing fluid particles ≥5 μm in diameter. Droplet size is highly variable and dependent on the force and pressure of emission, environmental variables including temperature, humidity, and airflow, the time spent airborne, turbulence, and the size of the pathogen within a droplet. As droplets evaporate, the remaining dried residue, or droplet nuclei, are often referred to as respiratory aerosols (<5 μm in diameter). While these size ranges are traditionally discussed as a dichotomy, research is showing that these particles are expelled and behave on a spectrum; particles at the larger end of the spectrum (≥5 μm in diameter) generally settle out of the air relatively quickly and close to their source (<1 m), while smaller aerosols (<5 μm in diameter) evaporate before settling, and the residues may remain airborne for much longer and more variable periods of time. Different types of masks, including disposable surgical masks and N95 FFRs are designed as protection against different types of potential exposures, and serve different purposes (2, 3).

Disposable surgical masks are designed to provide a one-time physical barrier to liquid droplets such as respiratory droplets ≥5 μm in diameter or blood during medical procedures, and are certified for this purpose by the FDA. They are designed to both protect the wearer from environmental liquids, and the environment from the wearer's respiratory secretions. While surgical masks do incidentally block some very fine particles <5 μm in diameter, including those containing airborne pathogens, their efficacy against these finer particles is unpredictable due to their loose fit, variable thickness, and variable materials used in construction; surgical masks should not be relied on for this purpose. Respirator masks may either have a valve or may be elastomeric, and are designed to form a seal around the nose and mouth in order to filter out airborne particles down to 0.3 μm in diameter including bacteria, viruses, and other physical or chemical irritants from the air breathed in by the wearer. N95 FFRs are named to indicate that they filter ≥95% of 0.3 μm Non-oil test particles under test conditions (4). This particle size is larger than most virus particles, including the influenza virus (0.06–0.1 μm) and coronaviruses (~0.125 μm) (4–7). Despite this size disparity, virions are generally contained within larger aerosols (1–5 μm in diameter) which disperse on contact with the filter of the respirator mask. The electronegative layer of disposable N95 FFRs serves to repel these fine particles from the wearer upon dispersal. To highlight this difference in design and function between surgical masks and N95 FFRs, one study found that the N95 FFRs tested filtered ≥99% of particles ≥0.1 μm, while the surgical masks tested only filtered 64–83% of particles ≥0.1 μm (8). As discussed above, these small particles (<5 μm in diameter) are often of greater infectious concern, as they remain airborne for longer periods of time (2, 3).

Within current veterinary practice, surgical masks and N95 FFRs are used in a wide variety of settings. FDA-certified disposable surgical masks are commonly used during surgical procedures to protect patients from bacterial contamination from the mask's wearer (9, 10). Similarly, surgical masks are worn during necropsy procedures, and during slaughter surveillance, to protect the wearer from any fluid droplets mobilized from cadavers (11). Surgical masks are also commonly worn during the routine care of swine, poultry, and non-human primates to protect against zoonotic viruses in droplets that may be emitted from those species such as influenza A and herpes B, and to similarly protect those animals from potential human diseases. The use of N95 FFRs is similarly commonplace. N95 FFRs may be used in all the above scenarios by individuals at increased risk, for instance elastomeric N95 FFRs may be worn by pregnant people concerned about anesthetic gas exposure during surgery, or exposure to potentially abortion causing zoonotic agents during necropsy. Similarly, those anticipating exposure to an allergen may preemptively wear a physical or elastomeric N95 FFR. N95 FFRs may be used during more invasive procedures with swine, poultry, or non-human primates (12–15). N95 FFRs may also be used with other PPE in the event of zoonotic outbreak and investigation, such as the Influenza A H1N1 Pandemic in 2009 or the Highly Pathogenic Avian Influenza A H5 outbreak in 2015 (15, 16).

### SARS-Cov-2 and the Need for Facemasks

With the current coronavirus disease (COVID-19) pandemic caused by SARS-CoV-2, all supplies of surgical and respiratory masks are severely depleted, in part due to the decreased production and disruption of the supply chain secondary to the effects of the pandemic on industrial workers, particularly in China, where this disease originated. Simultaneously, there is increased use of facemasks in response to a novel, deadly respiratory virus primarily transmitted via short-range, airborne respiratory droplets (1, 17). This increased use of facemasks is warranted based on existing English language literature regarding other viral respiratory infections with pandemic potential and similar transmission patterns, including SARS, MERS, and influenza (18–20). Moreover, recent studies of respiratory pathogen emissions in a small, turbulent cloud due to coughing indicate that, due to the fluid and air dynamics of the cloud, pathogens may be projected up to 7–8 m, and preserved by the localized humidity of the cloud. In light of these findings, facemasks may serve a further important function in limiting disease transmission by stopping such clouds from forming when the wearer coughs (3).

COVID-19 is very similar to Severe Acute Respiratory Syndrome (SARS); both diseases are caused by closely related betacoronaviruses, SARS-CoV-2 and SARS-CoV, respectively. Both diseases appear to follow similar modes of respiratory transmission, prolonged incubation and viral shedding periods, and cause similar symptoms, although they have different mortality rates (17–19, 21, 22). These similarities make studies on SARS excellent models for the response to COVID-19. Seto et al. performed a case control study of nosocomial infections in healthcare workers (HCWs) in Hong Kong hospitals during the SARS epidemic in 2003 and found statistically significant positive correlations between the proper use by HCWs of both surgical masks (*p* = 0.007) and N95 FFRs (*p* = 0.0004) and the prevention of nosocomial infections when all potential SARS exposures and index patients were known. It is worth noting that handwashing was also significantly correlated with the prevention of infection (*p* = 0.047), but the use of two layer paper masks was not protective (*p* = 0.511). The study also looked at the use of gloves (not preventative, *p* = 0.364) and gowns (preventative, *p* = 0.006), and found that no HCWs who utilized all 4 interventions appropriately, hand-washing, gowns, gloves, and facemasks (N95 FFRs when aerosolization was anticipated, surgical masks for droplet protection otherwise) were diagnosed with nosocomial infections (23).

Middle East Respiratory Syndrome (MERS) is a respiratory disease with a higher mortality rate than either COVID-19 or SARS, caused by another, slightly less closely related betacoronavirus, MERS-CoV. MERS was first reported in the Kingdom of Saudi Arabia (KSA) in 2012 (18). Over the following year, MERS was found to have a high rate of comorbidities in hospitalized patients similar to the comorbidities found with COVID-19 patients, including primary hypertension and diseases linked with secondary hypertension such as diabetes, cardiac disease, and kidney disease. Because of the influx of 2–3 million Muslim pilgrims to the KSA every year for the Hajj, immediate non-pharmaceutical measures were implemented including giving all pilgrims facemasks upon arrival to the KSA and emphasizing hand hygiene, modeled on interventions proven to be effective with SARS and influenza (24). However, the efficacy of these interventions implemented during a mass gathering on the scale of the Hajj is equivocal at best. In the wake of MERS, and given the known high rates of transmission of infectious respiratory diseases in general during the Hajj, Alfelali et al. undertook a 3-year cluster-randomized controlled trial. During the trial, pilgrims, clustered by tent, were given 50 surgical masks to wear continuously for the week of the Hajj. The pilgrims' respiratory symptoms and respiratory disease swab results following each Hajj were compared to a control group each year that was not given masks or extra instruction on PPE. Among the 7,687 participants, on an intention-to-treat basis, there was no significant difference in the incidence of either clinical respiratory disease nor in the incidence of laboratory-confirmed respiratory disease. However, it is worth noting that in the experimental arm of the trial, only 27% of 3,864 participants wore masks as directed, only 51% wore masks intermittently, and many members of the control brought and wore their own facemasks (15% continuous mask use, 38% intermittent mask use). Still, no association was found between facemask use and clinical respiratory illness within individual tents, either. Both arms of the trial had similar rates of frequent handwashing (~2/3) (25).

Similarly, in 2010 Aiello et al. at the University of Michigan conducted a cluster randomized controlled trial on students in residence halls studying the impact of wearing facemasks and maintaining hand hygiene on transmission of influenza. The researchers found a statistically significant (*p* <0.025) positive correlation between wearing surgical masks together with hand hygiene and the absence of influenza-like illness. Unfortunately, this particular study was underpowered and could not further parse out the effects of wearing a facemask from the effects of hand hygiene. However, this effect was seen in both the facemask only group and the facemask with hand hygiene group (26). In 2013 Milton et al. further found that surgical masks worn by influenza patients not only reduced droplet shedding of virus, but reduced aerosolized viral spread by those patients as well (27). Finally, early analyses by Cowling et al. (19) of preventive measures implemented in Hong Kong to control the COVID-19 pandemic in early 2020 also resulted in a significant decrease in cases of influenza, further supporting the translational nature of control measures recommended for influenza as valid against human coronaviruses.

Based on these and other studies, the WHO and CDC recommendations for the response to potential influenza, SARS, or other respiratory infection pandemics throughout the last twenty years have included use of surgical-style facemasks by patients with confirmed or suspected cases. These organizations have also recommended the use of N95 FFRs ± protective face shields or goggles when aerosols are expected, with front line HCWs using surgical masks otherwise (28, 29). With the current COVID-19 pandemic, most public health professionals recommend the general use of surgical masks by the entire population; because the dynamics of transmission and infection of COVID-19 are still poorly understood, and adequate testing has been lagging, they argue it is better for everyone to assume they are infected and wear masks to protect others until proven otherwise (30, 31). The CDC initially only recommended facemasks for HCWs due to significantly depleted supplies. This recommendation was made with the intention of funneling surgical masks to HCWs, so that health care systems in turn could funnel N95 FFRs to HCWs working directly with COVID-19 patients. However, the CDC updated its position in early April to recommend that the entire populace should be wearing homemade face coverings in public for the reasons outlined above (32, 33).

### Impact of Facemask Shortages on Veterinary Medicine

This funneling of all facemasks to the human health care system has greatly impacted other industries that routinely use these types of PPE, including veterinary medicine. Veterinarians are considered essential personnel in many states that have initiated varying degrees of shut-down orders, due to their roles in maintaining the food supply, supporting animal use in research including research to combat COVID-19, and providing essential emergency and palliative healthcare to companion animals. As evidence increases that wild and domestic animals, particularly cats, are also susceptible to COVID-19, veterinarians also have a growing role in not only treating animal patients with COVID-19, but also tracking disease spread, and limiting the spread of SARS-CoV-2 to an animal reservoir of yet unknown infectious potential to humans (34). Their continued ability to safely perform these vital functions is dependent on continued access to PPE, including facemasks. For companion animal practice, the largest sector within veterinary medicine, client interactions represent their highest risk of COVID-19 exposure, although there is also potential risk from infected pets (34, 35). However, there is little formal guidance on how to address facemask shortages

Anecdotally, social media and local news have seen a dramatic explosion of patterns for home-sewn fabric masks, and many human hospitals have embraced these patterns, asking for variations sewn with multiple layers of cotton, flannel, interfacing, etc. These requests are generally based on current CDC guidance that, given the severe shortage of FDA approved surgical masks and N95 FFRs, any physical barrier to personal droplets potentially carrying infective virions is better than nothing (36). The general public has latched onto making these masks as an action that can be undertaken while sheltering in place that seems helpful. Similarly, many companion animal practice veterinarians have been making and using these masks as stopgaps in their own practices. However, this reviewer was unable to find any validation for any of these patterns as physical barriers or respirator masks in the veterinary, medical, or other literature at the time of writing, despite various claims by pattern writers and pop news articles that these cloth masks can and do function for these purposes. Herein follows a review of relevant literature on mask use reduction, reuse, and the potential use of cloth masks in veterinary medicine. This reviewer was unable to find any studies specific to cloth or other alternative masks in veterinary medicine using search terms including various combinations of “cloth mask, face mask, facemask, mask alternative, facemask shortage, filtering face piece respirators, pandemic, COVID-19, personal protective equipment, SARS-CoV-2, history, surgical mask, Veterinary Medicine.” As such, relevant studies from the human medical literature are highlighted below, and all conclusions are necessarily drawn by analogy.

## Potential Changes to Facemask Use in Response to Shortages

### Disposable Facemask Use Reduction and Reuse

The most immediate course of action in the face of the pandemic is to reduce the use of facemasks in veterinary practices. This includes canceling elective and non-urgent procedures in which facemasks would be used, limiting the people involved in non-elective or urgent procedures who might need to wear a facemask, and potentially reconsidering whether a facemask is needed for each individual involved with a procedure (37, 38). Conversely, given mounting evidence that domestic and wild animals may also be infected with COVID-19, with poorly understood transmission potential to humans, it may be prudent for all veterinary professionals to wear facemasks when treating patients during this pandemic (34, 38). Finally, in the face of this human pandemic, human-to-human interactions in the pursuit of essential veterinary services, including client interactions, represent the most significant risk of COVID-19 infection for veterinarians and their staff, leading to an increased need for facemasks or other facial PPE in these routine situations (37, 38). The AVMA has recommended, based on the CDC recommendations, the use of expired facemasks, and also sanctioned limited extended use or reuse of disposable facemasks, as long as proper hygiene is maintained in donning and doffing masks, and there are no visible stains or tears. Proper hygiene includes limiting the use of any mask to a single wearer, avoiding touching the mask while it is being worn, washing hands before and after donning and doffing facemasks, and otherwise careful handling to avoid mask contamination (28, 32, 33, 36). Preexisting studies on how rapidly surgical masks or N95 FFRs decline in their designed function when reused in this way are limited, although the literature is currently expanding rapidly (39, 40).

Wearing a surgical mask, face shield, cloth mask, or some combination of these items over an N95 FFR may also help extend the function of the N95 FFR, although wearing multiple masks may negatively impact the wearer's ability to breathe easily. While face shields offer excellent droplet protection when worn correctly, they are limited by having open sides and bottoms. Particularly in the case of small animal surgery, this may still allow for droplets generated by the wearer to fall onto patients, and therefore face shields are not recommended as the sole facial barrier in this or similar situations. However, face shields may be ideal for certain client-facing interactions, particularly as many companion animal practices integrate curbside services to minimize human-to-human contact (41). Disinfection of disposable facemasks may not be feasible; liquid media such as bleach, alcohol, wet steam, and other cleaning agents render electronegative filtering membranes in surgical masks and elastomeric N95 FFRs ineffective, and degrade the barrier of the outer impermeable layer of these masks (42).

Fischer et al. have released preliminary research from the National Institute of Allergy and Infectious Diseases (NIAID) that indicates the use of vaporized hydrogen peroxide (VHP) or UV irradiation are preferred for sterilizing N95 FFRs with minimal impact on their function for up to three uses. These data indicate that VHP will inactivate enveloped viruses including SARS-CoV-2 faster than UV irradiation will, but that both methods are comparably effective when using appropriate protocols. Fischer et al. recommend using 70°C dry heat up to two times to resterilize N95 FFRs for reuse. They recommend against using ethanol, as their findings support previous research that ethanol inactivates N95 FFR filter membranes (43).

Fischer et al.'s findings are in line with recently released crisis standards from the CDC, which are based in part on these and other preliminary research around the country for sterilizing FFRs. The CDC standards cover a broader range of sterilization methods, similarly recommending VHP, UV irradiation, or moist heat, and suggesting that liquid hydrogen peroxide or microwave steam treatment may also be effective. The CDC standards are very clear that all other sterilization methods tested either changed FFR performance or function, including autoclave, dry heat, isopropyl alcohol, soap, dry microwave irradiation, and bleach. The CDC also mentions the use of disinfectant wipes, but their efficacy without altering FFR performance is questionable. While ethylene oxide treatment is likely effective, ethylene oxide is a known carcinogen and teratogen, raising the concern that FFRs sterilized with ethylene oxide may off-gas and injure their wearer. Finally, the CDC cites several recently published protocols that may be used as reference for recommended sterilization methods. However, many of these methods may not be available to most veterinary practices, necessitating the use of alternative facemasks. Moreover, Fischer et al., these new CDC standards, and the protocols and research they cite, are all in reference to FFRs and do not mention the effects of these methods on surgical masks that are much more commonly used in veterinary practice, nor on homemade facemasks that are increasingly being made and used, and therefore these protocols may not be transferable (43, 44). For these reasons, mask reuse is not the primary focus of this review.

### Disposable Facemask Alternatives

In their guidance on optimizing the supply of facemasks, the CDC states that “In settings where facemasks are not available, [HCWs] might use homemade masks for care of patients with COVID-19 as a last resort,” ideally in combination with a face shield that extends to or below the face and to both sides of the face (36). With the AVMA's endorsement and reiteration of these recommendations as applying to veterinarians, many veterinarians have applied this guidance to their practice, as well, and the CDC published specific guidance for companion animal veterinarians at the end of April (37, 38, 41). Particularly as many veterinarians donated their PPE to human health care providers prior to the release of evidence of COVID-19 infections in cats, the need for an alternative has become more urgent (34). What a homemade mask might be is very loosely defined, although several designs are now available on the CDC website, and the WHO just released guidance on the materials that should be used (28, 32, 33). AVMA, CDC, and WHO guidance are clear that homemade masks are not considered PPE, as their efficacy as barriers or filters is unknown, and that their use should be considered with “caution.” Early examples given were a bandana or scarf; more materials and designs are now sanctioned, but without data regarding the relative efficacy of those designs or suggested materials. While there have also been significant efforts mobilized for engineering 3D printed and other open source, reusable, respirator type masks and face shields, these items are outside the scope of this review.

Anecdotally, most requests for cloth masks and public efforts to donate cloth masks have focused on reusable cloth facemasks modeled on FDA-certified disposable surgical masks. Such masks are familiar to HCWs, and can be both laundered and sterilized for a constant supply in the face of shortages. There is an historical precedent for such masks in human medicine, and for adapting their use for veterinary medicine. However, there is a paucity of literature documenting the veterinary adaptation of facial PPE. In human medicine, and therefore presumably in veterinary medicine, predominantly cotton fiber cloth masks were the standard since the introduction of surgical masks in the mid-nineteenth century until the rise of disposable, fiber-blown mesh materials in the 1980s (45, 46). Cotton was the chosen fiber based on numerous studies conducted at the time, chosen over other fibers such as silk, nylon, or later polyester due to ubiquity, cost, comfort, ease of cleaning and sterilization, and inherent antimicrobial properties (47). One retrospective by Kool and Weinstein on preventive measures against person-to-person transmission of pneumonic plague, a zoonotic disease caused by *Yersinia pestis*, noted that during the 1920–21 Manchurian epidemic, physicians wore masks composed of half-inch thick cotton pads sandwiched in cotton gauze that extended into two sets of ties, and changed masks after each visit to the plague ward. This practice likely contributed significantly to the very low infection rate among physicians. Similarly, during a 1924 plague outbreak in Los Angeles, doctors and nurses protected themselves with masks made of celluloid and cotton, along with other PPE such as gowns and gloves, and no nosocomial transmission occurred (48). Incidentally, the cotton pad and cotton gauze masks used during the Manchurian epidemics are cited as early precursors to modern N95 FFRs (49).

These studies also largely support the current pleated design that most FDA-certified disposable surgical masks inherited for its ability to fit a variety of face sizes and shapes well and completely. One of the last studies to include a cotton mask, published by Quesnel in 1975, found that, of 5 masks tested, a reusable triple-pleated, four layer cotton muslin mask held in place by a pair of fabric ties was as effective a barrier as two other similarly pleated three layer early disposable masks, one made of two outer cellulose layers and an inner polypropylene layer, and the other made of two outer bonded rayon layers with an inner glass fiber layer. These three masks were all more effective than two other disposable masks tested. All three of the more effective masks were ~89% efficient as barriers to small particles (<3.3. μm), and >99% effective as barriers overall against the particle sizes measured (47). However, both testing and the tools to analyze the results of these types of studies have improved significantly since this particular study was done, and these results must be interpreted with those advances in mind.

More recent studies have also tested the efficacy of some models of homemade masks against modern disposable surgical masks. In a nearly prescient study published by the National Institute of Occupational Safety and Health (NIOSH) in 2010, Rengasamy et al. anticipated the potential shortage of disposable FFRs in the face of a viral respiratory pandemic such as SARS or influenza A, and a widespread rise in the use of masks made of common fabric materials in response (50). They specifically compared five major household fabric categories (3 sweatshirt fabrics, 3 t-shirts, 3 towels, 3 scarves, and 3 commercially available cloth masks) to N95 FFRs as filters against polydisperse (mixed size) and monodisperse (uniformly sized) NaCl aerosols 0.020–1 μm in diameter, using a similar NIOSH protocol to the NIOSH protocol currently cited in the recent FDA standard enforcement document for homemade FFR testing (5). It is worth noting that the cloth masks used in this study were commercially made and marketed as protection against pollution and allergens, without claims about their effectiveness against particles <1 μm in diameter, even though Rengasamy et al. were specifically investigating particles <1 μm in diameter. Similarly, the commercial cloth masks in this study may not be comparable to homemade cloth masks being made during the current pandemic; more information on fiber composition, fabric qualities, and mask design would be necessary for further comparison.

Rengasamy et al. tested their chosen fabrics three times each, in single layers, in tests run in parallel to a disposable N95 FFR using NIOSH protocols and NIOSH particulate respirator certification equipment. Each material was tested at two air speeds, 33 and 99 L/min, to simulate normal breathing and higher velocity emissions (e.g., sneezing or coughing), and data were collected on both particle penetration and the pressure drop across a single layer of each fabric. Overall, the masks and other fabrics ranged from 40 to 90% polydisperse aerosol penetration, and 9–98% monodisperse aerosol penetration at both air speeds, relative to 0.12% penetration of the N95 FFR at 33 L/min and <5% penetration of the N95 at 99 L/min. Rengasamy et al. also tested the pressure drop across each of the materials as a metric for ease of breathing, and found that all of the fabrics had pressure drops less than or comparable to that seen across the N95 FFR (9.8 cmH_2_O at 33 L/min). They also compared these findings to a previous study led by Rengasamy of 5 different commercially available disposable surgical masks and found that the sweatshirts and one of the scarves had penetration values comparable with FDA-certified surgical masks. However, this study only evaluated these fabrics against aerosols <1 μm and did not compare their efficacy as a barrier to larger droplets or other liquids relative to surgical masks. Moreover, Rengasamy et al. were unable to define any specific fiber composition (cotton, polyester, or a blend) or fabric structure (woven, knit, non-woven) that might lead to decreased particle penetration, and therefore be a more appropriate choice for a homemade face covering. Overall, Rengasamy et al. concluded that face coverings made of homemade fabrics offered only marginal respiratory protection from particles <1 μm, but were likely better than nothing in the case of a potential viral respiratory pandemic and shortage of appropriate FFR (50).

In 2014, Jung et al. in South Korea subjected 44 different commercially available masks along with bandanas and gauze in 1–4 layers to both Korean Food and Drug Administration (KFDA) and NIOSH FFR testing. The masks were of various grades including KF94 masks (the Korean equivalent of N95 FFRs), KF80 masks that filter out 80% of aerosols [NaCl was used in this test, similar to (50)], surgical masks, and what the group referred to as “general” masks. It is worth noting that there were both KF80 masks and surgical masks made from cotton on the market in South Korea. However, the design of these masks was not readily available for comparison. Still, these cotton KF80 masks (76.279% filtration, *p* = 0.0029) and surgical masks (41.625% filtration, *p* = 0.9459) performed comparably in testing with non-woven masks in the same categories. The large *p*-value of the surgical masks reflects the broad performance range of the various masks in that category within the study (51).

Jung et al. broke the general masks into non-woven and cotton masks, with the non-woven masks filtering significantly (*p* = 0.0004) more aerosols (54.750% using the NIOSH protocol) than the cotton masks (22.633%). Of the homemade options tested, four layers of cotton handkerchief performed the best, filtering out 3.800% of aerosols (*p* = 0.0013), with four layers of gauze handkerchief coming in a close second (3.633%, *p* = 0.0001). The group also tested the pressure drop across each of the masks as a metric for ease of breathing. While all the masks tested using the KFDA protocol were within the KFDA standard (pressure drop <7.2 mmH_2_O for KF94 masks, <6.2 mmH_2_O for KF80 masks), four layers of cotton handkerchief had the second highest pressure drop at 3.433 mmH_2_O (*p* <0.0001), while four layers of gauze handkerchief were more in line with most of the other masks with a pressure drop of 2.967 mmH_2_O (*p* <0.0001) (51). These findings suggest that homemade cloth masks, especially in the two layer designs currently popular, are in no way a replacement for N95 FFRs. However, without more information about the fabric density and construction of the cotton masks and handkerchiefs included in this study, it is difficult to compare these masks to surgical masks, particularly given the filtration of all surgical masks grouped together was not significant.

At Cambridge the same year Davies et al. used similar filtration and pressure drop tests to assess the suitability of materials commonly found in the home for making surgical-style masks, as well as one homemade mask design, in the event of an influenza pandemic and similar PPE shortage as is now being seen with COVID-19. Instead of aerosolized NaCl, Davies et al. used a mix of non-pathogenic virus and bacteria that covered the range of influenza virion size (0.06–0.1 μm; the microbes used covered a range of 0.023–1.25 μm) (6). Conveniently, influenza virions are similar in size to coronavirus virions (7). This range also encompasses the sizes of other microbes of veterinary interest, including *Yersinia pestis, Bacillus anthracis, Francisella tularensis*, and *Mycobacterium bovis*. While the group found that a vacuum cleaner bag (94.35% bacterial filtration, 85.95% viral filtration) and two layers of tea towel (96.71% filtration of bacteria, 72.46% one layer viral filtration; two layer viral filtration not listed) had filtration efficiency approaching that of the surgical mask (96.35% filtration of bacteria, 89.52% filtration of virus), the pressure drop across these materials (vacuum cleaner bag = 10.18 mmH_2_O, two layers of tea towel = 12.10 mmH_2_O) was far in excess of that across the surgical mask (5.23 mmH_2_O) (6).

Because Davies et al. was trying to design a mask the general populace might tolerate, they designated t-shirt fabric or pillowcase fabric the most appropriate materials for a two layer surgical-style facemask, with two layer bacterial filtration of 70.66 and 62.38%, respectively, one layer viral filtration of 50.85 and 57.13%, respectively, and pressure drops across two layers of 5.13 and 5.50 mmH_2_O, respectively. The group further subjected their two layer t-shirt fabric homemade mask to respirator fit testing and compared the results to respirator fit testing of their surgical mask. Davies et al. quantified their fit testing results as a fit factor, defined as the ratio of the number of microscopic particles measured outside each mask to the number measured inside each mask. While the surgical mask performed significantly better than the homemade mask in all tests (*p* <0.001), the homemade mask was able to reduce the number of pathogens expelled when coughing significantly relative to the no mask control (*p* = 0.004). As such, Davies et al. concluded that the homemade mask was better than no mask, but should be “viewed as the last possible alternative” in the face of a shortage of PPE (6).

Davies et al. was building on previous work done by van der Sande et al. in the Netherlands in 2008. The Dutch group used fit testing to compare an FFP-2 respirator mask (filtering facepiece 2; the European equivalent of an N95 FFR) to a surgical mask and a single layer homemade surgical-style mask made from a tea cloth, in another effort to test facemask options for the general population in the case of an influenza pandemic. Notably, van der Sande et al. cited the contribution of population-wide use of facemasks during the SARS epidemic in Asia to the reduction of influenza cases during that time as part of the inspiration for their study. They performed both short-term (15 min) and long-term (3 h) tests with volunteers wearing each mask while performing different activities to test how well the masks protected the volunteers, using probes placed on the inside and outside of each mask to measure particle concentration differences across the masks while they were being worn. Finally, they used a manikin to simulate various respiratory flow rates, using the probes from the previous experiment to measure how well the masks acted as barriers to particles generated by the manikin (52).

All the homemade masks in van der Sande et al.'s study provided protection to their wearers both in the short and the long term; the group calculated the FFP-2 respirator mask to be ~25 times more effective at protecting the wearer than the surgical mask, and ~50 times more effective than the homemade tea cloth mask. On the manikin, they found that all masks were less effective as a barrier to particles generated by the wearer than they were at protecting the wearer; they found the FFP-2 respirator mask and the surgical mask did not differ and were roughly equally protective, but that the homemade mask was only marginally effective as an outward barrier. In line with the other groups, van der Sande et al. concluded that these homemade masks can still decrease viral transmission, and remain effective as protective barriers over longer periods of time (52).

Separately, Dato et al. in Pittsburgh devised a cloth mask using nine layers of heavyweight t-shirt material with three sets of ties, in an attempt to mimic an N95 FFR using only materials most people would have readily available. They based their material choice on heavyweight, two-ply t-shirts previously experimentally used to protect mice from ricin and saxitoxin. The Pittsburgh group used quantitative fit testing designed to fit N95 FFRs to measure the efficacy of their hand-fashioned respirator mask. N95 filtering facepiece respirators are expected to achieve a fit factor of 100 in order to be effective using this test; one individual achieved a fit factor of 67 with the hand-fashioned respirator mask, the other two volunteers achieved fit factors of 13 and 17. All volunteers were equally able to breathe through the hand-fashioned respirator masks and fitted N95 FFRs. Overall, Dato et al. concluded that hand-fashioned masks can offer good fit and measurable protection against aerosols in the absence of commercially available masks, but that commercially available masks were preferable. Again, this group was not testing their mask as a surgical barrier, but as respiratory protection for the wearer against pathogens such as avian influenza A H5N1 (53).

Notably, MacIntyre et al. conducted a randomized clinical trial with nurses in Hanoi to compare the efficacy of cloth masks and disposable surgical masks. The group concluded that wearing cloth masks was worse than wearing nothing for nurses caring for those with infectious respiratory diseases. However, the trial did not include a negative control, as telling nurses not to wear any mask was rightfully deemed unethical by the Institutional Review Board overseeing the study. The three trial arms were surgical masks, cloth facemasks, and a control of the nurses' normal practice, which was largely to wear surgical masks. Only two study participants out of 458 in the control arm reported using no facemasks; 53% reported wearing both surgical and cloth facemasks, 37% reported wearing only surgical masks, 8% reported wearing only cloth facemasks, and <1% (3 individuals) reported wearing only N95 FFRs. When investigators instead looked at infection rates between those who only wore surgical masks and those who only wore cloth facemasks across all three study arms, those who only wore cloth facemasks had a significantly higher rate of respiratory infections. However, no other hygiene interventions were mentioned, so confounding factors such as hand hygiene, or how often cloth masks were sterilized or replaced, if at all, were not accounted for in this study. Overall, MacIntyre et al.'s study shows that cloth facemasks are not as effective as disposable surgical masks assuming all other factors are equal. These data cannot unequivocally indicate that wearing nothing is better, since a no-mask arm was not included in the investigation, despite the groups' stated conclusion (46).

## Discussion

### Currently Circulating Designs

During the current COVID-19 pandemic, the studies cited above, along with a few others that show similar results, have been cited in the design of multiple homemade masks. Many designs mimic the pleating of surgical masks; however, discussion is largely centered on mimicking the efficacy of N95 FFRs. To that end, pocketed designs have been popular to allow the use and replacement of filters approaching the efficacy of an N95 FFR. Myriad materials have been proposed as filters, including vacuum cleaner bags based on the Davies et al.'s findings, high-MERV (minimum efficiency reporting value, measured on a scale from low to high of 1–20) or HEPA (high-efficiency particulate arrestance, equivalent to MERV 17–20) HVAC (heating, ventilation, and air conditioning) filters due to their fine filtering ability, and items such as disposable blue shop towels, diapers, and menstrual pads (54, 55). These specific filters are less than ideal. As cited by Davies et al., vacuum cleaner bags are rigid and difficult to mold to the face, limiting their ability to function as an appropriate barrier (6). Moreover, many vacuum cleaner bags and HVAC filters are made with fiberglass or other potentially harmful materials that may pose a separate health risk to the wearer, and therefore are not recommended (56). The gaining popularity of high-MERV HVAC filters is particularly concerning, as these filters are rated efficient assuming preservation of many layers of material, but many designs only call for the use of a single layer. Similarly, the disassembly of these filters for use exposes one to hazardous materials with risk for injury, and the high proportion of “hot melt adhesive” on 3M's Material Safety Data Sheet (MSDS) for their MERV 12 Filtrete filter may indicate the filter material is intolerant of high heat (57).

Disposable blue shop towels have recently been put forward as reasonable mask or filter material, based on purported N95 testing of one design with unknown controls or environment (58). A product technical bulletin from Kimberley-Clark, the makers of Scott blue shop towels, indicates they are relatively inert, however, they are highly absorbent, which limits their utility as a protective barrier, at least until the effect of increased moisture on their filtering ability is better understood. Moreover, the high cellulose content of blue shop towels makes them incompatible with any sterilization protocol using hydrogen peroxide, which will degrade cellulose (5, 59). Both diapers and menstrual pads are made to be absorbent and impermeable, qualities at odds with the continued respiration of the wearer. Moreover, with impermeable barriers, wearers have been finding, anecdotally, that they take in air around the edges of the mask, defeating the purpose of the mask as a filtering barrier during the COVID-19 pandemic. Finally, none of these filters are designed for prolonged proximity to mucous membranes and may have unexpected toxicities, fumes, or other issues that may be detrimental to the wearer.

There are a few promising options. Segal et al. at Wake Forest Baptist Medical Center have indicated that they have made two layer cotton cloth masks that are comparable with disposable surgical masks out of densely woven batik quilting fabrics, similar to the results seen in Jung et al. described above, although Segal et al. have not published their designs nor data (51, 60). Another option may be to make surgical-style masks out of medical textiles including pack wrapping material, surgical drapes, etc., which are made to be autoclaved, impermeable to fluids, but permeable to steam so that the contents of surgical packs may be sterilized. Spiess et al. at the University of Florida at Gainesville seem to be having some success with Halyard H600 medical fabric, which is normally used as pack wrap; however, they have not published their data, and early anecdotal reports indicate masks using their design and materials are difficult for lay people to breathe through. Spiess et al. also appear to have used N95 FFR fit testing equipment to assess the efficacy of their Halyard H600 homemade mask, similar to the studies above, which is promising, but difficult to interpret without access to the results of that fit testing (61). It seems likely that similar materials will have similar efficacy; while Speiss et al. specifically used Halyard H600 medical fabric, Meijer and Vrielink in the Netherlands have released preliminary data validating two similar designs using Halyard Quickcheck H300. Meijer and Vrielink's first design has three layers and fit-tested as equivalent to FFP-2 on first use, and equivalent to FFP-1 after steam sterilization for 5 min at 135°C. Their second design is constructed using two layers of Halyard Quickcheck H300 and is initially equivalent to FFP-1 (62). Moreover, a proposal from Gadi et al., a group collaboration between Boston Medical Center and Boston University School of Medicine, suggests that other medical textiles, such as the trilaminate fabric used in dry suits, may be even more suitable as breathable fabric for a reusable mask, although the group did not address whether this material may be autoclaved or otherwise sterilized for reuse (55).

Similarly, Kwong, a chemistry professor in Hong Kong, publicly released a design called the Hong Kong Mask, which is a cloth pocket mask that uses facial tissues or paper towels as filters. There are infographics available in multiple languages on the design website showing both filtration efficiency of particles 0.3 μm in diameter and the pressure drop across the mask with 1–3 filtration layers in the mask using different potential orientations. The mask with two paper towels perpendicular to each other as a filter is reported to filter 91.30% of particles. However, the pressure drop is 66.4 mmH_2_O with air flowing at 32 L/min, which indicates this may be difficult to breathe through. A three layer filter of facial tissues, all oriented the same way, is roughly as effective as the surgical masks in the above studies, with a filtration efficiency of 83.00%, and much more breathable, with a pressure drop of 24.8 mmH_2_O; this is still roughly 4 times more difficult to breathe through than a disposable surgical mask, and a greater pressure drop than Davies et al. reported for their two layers of tea towels (6). The author of this review was also unable to find the actual study data that went into these infographics, so it is difficult to determine whether these results are comparable to the studies above. Further, no information is given for if the filters become wet and start degrading, a real concern for veterinary use as a barrier against droplets (63).

Both Davies et al. and van der Sande et al. noted that “tea towels” or “tea cloths” had reasonably high filtration efficacy and breathability (6, 52). However, neither group defined the properties of the material for reproducibility. While van der Sande et al. did state that they used a specific tea cloth made by the company Blokker, that cloth no longer appears to be available, and without information on thread counts, grams per square meter (GSM) and fiber, it is unknown how similar other tea cloths on the website may be (64). Davies et al. included even less information. A search of the internet of things revealed that tea towels may be similar to kitchen towels, and that a standard thread count for tea towels may be 130 thread count (65). Two hundred and seventy thread count pima cotton is considered standard for reusable surgical drape and other medical linens in veterinary medicine; it may be prudent to instead defer to that more conservative standard (66, 67).

### Conclusions and Recommendations

Many of these studies assess the efficacy of homemade masks relative to the filtration achieved by N95 FFRs. Particularly in human medicine, unlike veterinary medicine, the N95 FFR has become the standard of clinical care as the most conservative type of respiratory PPE in the face of communicable disease that is almost always contagious to HCWs. In companion animal practice, the largest single sector of employment within veterinary medicine, most patient infectious disease is not a zoonotic concern, although concern that COVID-19 infected animals may be able to transmit disease back to humans may be reasonable (34, 35, 38). Because of this, the risk profile for the majority of veterinarians and their support staff is usually significantly different from that of human HCWs. Face masks are generally used in clinical practice as a barrier to protect the wearer from larger droplets (≥5 μm in diameter) and liquids and to protect surgical patients from nosocomial infections, and the literature must be read with this purpose in mind.

While the inclination might be to mimic the most conservative option (N95 FFRs), particularly as veterinarians also face a global human pandemic to which they are personally susceptible, the literature indicates that disposable surgical masks or homemade facemasks to arrest the respiratory emissions of veterinary staff and to protect animal patients are largely adequate in the clinical setting, although every practitioner should evaluate their personal risk individually. Moreover, the literature indicates that N95 FFRs do not maintain their seals, and therefore their filtration capacity, when worn over extended periods of time. In contrast, van der Sande et al., the one study that tested all three types of masks over an extended period, found that the cloth mask maintained efficacy over the duration of the experiment (52). Finally, compliance is an issue in all of the trials that involved people wearing masks over multiple days. Alfelali et al. in particular found that breathability was a major factors in whether study participants wore masks as prescribed (25). In fact, more breathable surgical or cloth mask designs may be preferable to N95 FFRs or masks made from medical materials that pass fit-testing as equivalent to N95 FFRs for this reason.

As such, instead of true FFRs, in the absence of potential aerosols <5 μm in diameter carrying zoonotic risk, veterinarians, and perhaps the general public, should focus on substitute barrier masks (38). Most importantly, veterinarians must also continue practicing appropriate hand hygiene, which all data indicate to be the single most effective intervention against all infections, including surgical site infections, and social distancing as much as possible (10, 37, 38, 66–68). The above studies largely focused on appropriate homemade barrier facemasks for the general population. However, veterinarians are already trained in the use of less breathable, more effective masks including N95 FFRs. Due to their increased training, combined with the real risk of surgical site infections and possible risk of zoonotic COVID-19, at the time of this writing Davies et al.'s two layer tea towel mask or Segal et al.'s two layer quilting cotton mask, or a similar mask using two layers of densely woven (≥270 thread count) pima cotton or similar medical textile may be most appropriate, as they most closely approximate the bacterial and viral filtration efficacy of surgical masks along with maintaining moldability for a close fitting barrier (6, 60). The WHO recently recommended constructing masks out of three layers that mimics the construction of disposable surgical masks and N95 FFRs, using a hydrophobic polyester outer layer, electronegative interfacing middle layer, hydrophilic cotton inner layer closest to the wearer. While this mask has not yet been tested as a final product at the time of this writing, it may represent another appropriate option, or at least suggest a reasonable filter (interfacing) to use with two layer tea towel or cotton fabric masks (28). Other types of cloth or non-woven materials and other mask designs may also be effective, as the current research is progressing daily.

As a general guide to optimizing homemade, reusable facemask design based on the designs reviewed here and elsewhere, masks should have enough surface area to fully cover the lower half of the face, including both the nose and mouth, and extending to the sides of the face beyond the corners of the mouth (42). Pleating or gathering along the sides may help ensure a good fit across a variety of face types. While more fitted, three dimensional designs are available, they require more customization in cutting and sewing to fit a wearer well and may take longer to make. A piece of jewelry wire, craft wire, or pipe cleaner may be included in the top hem to help fit the mask over the nose; cloth tape such as Elastikon may also be used to hold the top of a mask to the face and nose (61). While many patterns currently circulating use elastic over the ears, veterinary practitioners are likely to be more familiar with two sets of ties, which allow for a longer lasting, more customizable fit, and which are more durable in the face of typical veterinary laundering and autoclaving practices. If a densely woven fabric, appropriate medical textile, or other PPE material is not available, the Hong Kong Mask may be a reasonable alternative, using materials designed for proximity to mucous membranes as filters (63). Consider making the outside and inside layers of the mask visibly different so that they are easily distinguishable, to aid in minimizing handling and maintaining mask hygiene. Homemade facemasks should always be laundered and sterilized, if possible, before use.

If they have the equipment readily available, veterinarians should consider fit testing all homemade masks using an FDA-certified disposable surgical mask as a control for comparison. While these masks are not FFRs, such testing will allow individual veterinarians to assess whether the design they have chosen will meet their barrier and filtration needs in reference to the surgical mask standard to which they are accustomed. Ideally, fit testing will be over a long enough duration to assess whether absorption of moisture from the wearer's breath, perhaps during the course of a surgical procedure, is an issue. As designs that achieve filtration efficiencies closer to those of N95 FFRs become available, if their use as an FFR is desired, consider testing such masks according to the NIOSH procedures outlined in the FDA Enforcement Policy for Face Masks and Respirators During the Coronavirus Disease (COVID-19) Public Health Emergency (5). Practices should strive to make enough masks that they are able to change masks as frequently during a normal day as they would using FDA-certified disposable surgical masks, as continuous use of a cloth mask has at least the same potential for the mask acting as a fomite that a surgical mask carries (69). Homemade facemasks should be laundered and sterilized with other practice linens. Finally, pending further research, as recommended by the AVMA, CDC, and WHO, the substitution of homemade masks for FDA-certified surgical masks should only be considered as a last resort, until and unless the FDA certifies a reusable homemade mask or design. Consider the above guidelines for extended use, reuse, and resterilization to extend the current supply of masks, or even the use of a single layer cloth mask over a surgical mask to help keep the surgical mask clean and further extend its useful life (28, 37, 38, 41).

## Author Contributions

AP contributed conception, research, wrote the first draft of the manuscript, contributed to revision, read, and approved the submitted version.

### Conflict of Interest

The author declares that the research was conducted in the absence of any commercial or financial relationships that could be construed as a potential conflict of interest.

## Abbreviations

CDC: Centers for Disease Control and Prevention
COVID-19: Coronavirus Disease 2019
FFP: Filtering FacePiece
FFR: Filtering Facepiece Respirator
GSM: Grams per Square Meter
HCW: Health Care Worker
HEPA: High-Efficiency Particulate Arrestance (equivalent to MERV 17–20)
HVAC, Heating, Ventilation: and Air Conditioning
KSA: Kingdom of Saudi Arabia
KFDA: Korean Food and Drug Administration
MERS: Middle East Respiratory Syndrome
MERV, Minimum Efficiency Reporting Value: on a scale from low to high of 1–20
NIOSH: National Institute of Occupational Safety and Health
PPE: Personal Protective Equipment
SARS: Severe Acute Respiratory Syndrome
SARS-CoV-2: Severe Acute Respiratory Syndrome Coronavirus 2
VHP: Vaporized Hydrogen Peroxide
WHO: World Health Organization
AVMA: American Veterinary Medical Association
NIAID: National Institute of Allergy and Infectious Diseases
MSDS: Material Safety Data Sheet.
